# Comparative evaluation of dissolution profiles of the generic drug lamivudine 150 mg tablet marketed in Peru vs. the innovative Epivir

**DOI:** 10.17843/rpmesp.2024.411.12821

**Published:** 2024-03-25

**Authors:** Malena Castañeda-Alarcón, Encarna García-Montoya, Javier Rodríguez-Calzado, María Flores-Rodríguez, Miguel Grande-Ortíz, Luis Moreno-Exebio

**Affiliations:** 1 National Center for Quality Control, National Institute of Health, Lima, Peru. National Center for Quality Control National Institute of Health Lima Peru; 2 Department of Pharmacy and Pharmaceutical Technology and Physical Chemistry, Faculty of Pharmacy and Food Sciences, University of Barcelona, Barcelona, Spain. University of Barcelona Department of Pharmacy and Pharmaceutical Technology and Physical Chemistry Faculty of Pharmacy and Food Sciences University of Barcelona Barcelona Spain

**Keywords:** Therapeutic Equivalence, Generic Drugs, Lamivudine, Bioequivalent Drugs, Dissolution, HIV

## Abstract

Lamivudine is one of the most prescribed drugs in the world, and is used to treat human immunodeficiency and hepatitis B. This study aimed to evaluate the quality attributes and compare the dissolution profiles of two batches (A and B) of generic lamivudine 150 mg tablets with the innovator drug Epivir 150 mg tablets. We conducted an analytical, experimental, cross-sectional study, and used a spectrophotometric method at a wavelength of maximum absorption (λ) corresponding to 270 nm, to measure the percentage of dissolved drug. The study evaluated identification, content, dissolution and mass uniformity. Apparatus 2 USP (Paddle) 75 rpm, 900 mL of dissolution medium (37 ± 0.5 °C) was used in three dissolution media: pH 1.2; 4.5 and 6.8. Samples of 5 mL were obtained at 5, 10, 15, 20 and 30 min. Both batches of generic lamivudine (A and B) were found to have the same dissolution kinetic profile as the innovator drug. Both formulations met the criteria of very fast dissolving (85% dissolved in 15 min), and fast dissolving (85% dissolved in 30 min) drugs. Therefore, it was not necessary to calculate the similarity factor. We concluded that generic drugs A and B are in vitro equivalents to the innovator drug Epivir.

## INTRODUCTION

Human immunodeficiency virus (HIV) infection is a major public health problem in the world and antiretroviral treatment prevents HIV-related illnesses. In Latin America, only 65% of cases receive treatment; in Peru ^1^^,^^2^ the National Program for Highly Active Antiretroviral Therapy (HAART), through the Peruvian Ministry of Health (MINSA), works to control the HIV epidemic through prevention strategies, providing treatment free of charge, ensuring medication availability and adherence ^3^.

Ensuring that drugs meet quality standards is mandatory to guarantee the safety and efficacy of the drug. A generic or multisource drug (MD) is pharmaceutically equivalent (alternative) and may or may not be therapeutically equivalent to the innovator (reference). Only a MD that has demonstrated therapeutic equivalence to the innovator (*in vivo* or *in vitro*) is interchangeable ^4^. In the European Union and the United States of America, therapeutic equivalence is a regulatory requirement for generic drug registration ^5^.

Interchangeable generics are marketed in Mexico, Brazil and Argentina; however, in Bolivia, Honduras, Nicaragua, Dominican Republic and other countries such as Peru, pharmaceutical products are still marketed without therapeutic equivalence studies ^6^. In Peru, for the registration, inscription and re-inscription of drugs, the regulatory authority requests interchangeability studies through Supreme Decree 024-2018-SA, which is being applied in the country gradually or progressively, its first list included lamivudine (LMV) tablets, for which *in vitro* studies (bioexcencion) are accepted ^7^.

A drug must meet pharmacopeial requirements to demonstrate its quality, including the dissolution test that determines the percentage (%) of drug dissolved in a single point. To demonstrate *in vitro* therapeutic equivalence, in addition to the solubility and permeability of the drug, the similarity of the dissolution profiles (dissolved in several points) is evaluated, and the MD must show a behavior similar to that of the innovator. However, there are occasional reports of generic drugs of inferior quality worldwide ^(^^8^. In Peru, generic drugs that did not meet the required quality were found, among them, some batches of metformin hydrochloride 850 mg ^9^, moxifloxacin tablets 400 mg ^10^^)^ did not pass the dissolution profile similarity test. For this reason, it is imperative to investigate the quality of the drugs dispensed in the country, in this case LMV, a drug for the treatment of HIV provided by the HAART program of MINSA.

This study aimed to evaluate the quality and compare the similarity of the dissolution kinetics of two batches of MD LMV 150 mg tablets, from the HAART program of MINSA, through dissolution profiles compared to the innovative drug (reference) Epivir.

**KEY MESSAGES**

Motivation for the study: To evaluate the quality of antiretroviral drugs used in the treatment of HIV dispensed in the HAART Program of the Ministry of Health of Peru.

Main findings: Two batches of generic lamivudine drugs were found to achieve a dissolution rate greater than 85% at 15 min, being equivalent in vitro to the reference product Epivir.

Implications: There is a need to apply the current regulations regarding equivalence between drugs by the regulatory authority prior to their authorization and to include dissolution profile tests as a requirement in public drug purchases, especially in national strategies (HIV, TB, etc.), in order to ensure quality products for the population.

## THE STUDY

The list of chemicals and reagents is available in the supplementary material.

### Type of study and samples

The was an experimental study, conducted at the Drug Development Service (SDM) of the University of Barcelona, and at the National Quality Control Center (CNCC) of the INS, the sample consisted of 200 tablets of the 150 mg LMV innovator drug and 200 tablets of 150 mg LMV MD from batch A and B dispensed by the HAART program of MINSA in 2019 (Table 1).

**Table 1:** Manufacturing information of the evaluated drugs.

Information	Active ingredient		
	Reference	Batch A	Batch B
	Epivir (lamivudine)	Lamivudine	Lamivudine
Declared quantity	150 mg	150 mg	150 mg
Presentation	Tablets	Oral tablet	Oral tablet
Batch	275 P	E180608	E182268
Country of origin	United Kingdom	India	India
Manufacturer	Glaxo	Hetero Labs Limited	Hetero Labs Limited
Expiration date	10-2021	03-2020	10-2020

### Method validation and equipment calibration

The dissolution profile was validated by spectrophotometric method. We considered the following parameters: linearity, precision, accuracy, stability and filter influence, according to United States Pharmacopeia (USP 42) International Conference on Harmonization (ICH) ^12^^,^^13^. For linearity, a calibration curve was prepared for seven concentrations in the range of 10% to 120% of the LMV standard (St). For accuracy, we analyzed 10%, 60%, and 120% St LMV solutions. For precision, six replicates (n=6) at 100% of LMV St were analyzed and the relative standard deviation (RSD) was calculated. For filter influence, 100% LMV St was used and filters of different materials and pore sizes were used. For stability, LMV St solution was read at 100% and stored at 2 to 8 °C for 24 h, then its concentration was measured again. All samples were read in triplicate at λ = 270 nm in the three-dissolution media (pH: 1.2; 4.5 and 6.8).

### Quality tests

LMV identification, content, dissolution and uniformity assays were performed according to USP 42, general chapters <197M>, <621>, <711> and <905>. Drug identification was performed by Infrared (IR) spectrometry method and by ultraviolet-visible (UV-VIS) high performance liquid chromatography (HPLC). The content assay by HPLC - UV-VIS with UV 277 nm detector; column L1 25cm × 4.6 mm (5 um), volume 20 uL, flow rate 1mL/min and column temperature 30 +/- 5 °C. Dissolution by UV-VIS spectrometry and uniformity of dosing units was performed by weight variation ^12^. 

### Dissolution profile

We measured the amount of drug released (percentage of the labeled amount). Six and 12 tablets were analyzed for batches A, B and the innovator. Samples were taken manually with replenishment (10 mL each time) at 5, 10, 15, 15, 20 and 30 min; subsequently 1 mL of the aliquots taken from each sample were made up to 10 mL in the three-dissolution media, filtered with 0.45 µm PVDF and the absorbances were read at λ = 270 nm by UV-VIS spectrophotometric method ^4^^,^^12^. All media were prepared without enzymes and degassed under vacuum with mechanical agitation. Assay conditions were apparatus 2 (paddle) at 75 rpm and 900 mL volume each dissolution medium at 37 ± 0.5 °C ^12^. The equipment used for dissolution profiles were Erweka® DT 700 dissolver for 8 vessels and Specord 205 Analitikjena spectrophotometer, Hanson Vision Elite G2 Heather dissolver for 8 vessels and Janson V-650 Spectrophotometer.

### Statistical analysis

Microsoft Excel 2019 was used as the primary tool. To enhance the functionality and accuracy of the evaluations, specialized add-ins were incorporated, including “tools for analysis” that provided advanced statistical functionalities. We used DDSolver, an Excel add-in, for the calculation of the similarity factor between drug dissolution profiles (f2), however, it was not applied because the drugs dissolved more than 85% in less than 15 min. The dissolved amount of the active ingredients was carried out using scatter plots, considering each batch and the pH corresponding to the dissolution profiles. Regarding the validation parameters, we applied descriptive statistics, such as the calculation of the arithmetic mean, standard deviation and relative standard deviation.

## FINDINGS

The validation results demonstrate that the method is linear over the concentration range of 0.0170 to 0.2037 mg/mL for all dissolution media. The values we obtained for the relative standard deviation (RSD) and recovery of standard solution samples indicate that the precision and accuracy of the method is satisfactory. The 0.45 μm PVDF filter type was selected because it had the highest % recovery among the evaluated filters, and the method was found to be stable at 24 h under refrigerated conditions (2 to 8 °C) (Table 2).

**Table 2:** Validation of the dissolution profile method.

Parameters	Specifications ^a^	Results		
		pH=1.2 ^b^	pH=4.5 ^c^	pH=6.8 ^d^
Linearity	r not less than 0.999	1.0000	0.9997	1.0000
	CV % less than or equal to 2%	0.366%	1.521%	0.478%
	Intercept	0.0022	- 0.0017	0.0006
Precision	DSR % less than or equal to 2%	0.03%	0.06%	0.02%
Exactitude	Recovery rate 95-105%	102.33%	103.13%	100.41%
	DSR % less than or equal to 2%	0.3882%	0.9575%	0.4077%
Stability	Recovery rate 98-102%	98.36%	98.80%	99.69%
Filter influence ^e^	Recovery rate 98-102%	99.05%	98.63%	94.27%

a Acceptance criterion, according to ICH Validation Of Analytical Procedures: Text And Methodology Q2(R1)

b Simulated gastric fluid pH 1.2

c Acetate buffer pH 4.5

d Simulated intestinal fluid pH 6.8

e The 0.45 μm PVDF filter showed a higher % recovery rate

r: Pearson’s correlation coefficient

CV %: coefficient of variation of the response factor, DSR %: relative standard deviation

### Drug quality control

The innovator drug and the two batches (A and B) of MD passed the quality tests: identification, content, dissolution and mass uniformity, as required by USP 42. For the identification of the innovator and MD batch A and B, the chromatograms showed that the retention times correspond to the standard LMV solution. The content of all samples was found to be within 90 to 110%. Regarding the dissolution tests, the percentage recovery of the innovator ranged from 102 to 103%, it was 97 to 105% for the MD batch A and 99 to 101%for MD batch B. All met the specification of dissolution ≥ 80% of the labeled amount. The uniformity of all samples was within the acceptance range of USP 42 (Table 3).

**Table 3:** Quality control results of innovator (reference), lamivudine batch A and B.

Testing	Specifications ^a^	Results		
		Reference	Multisource Batch A	Multisource Batch B
Lamivudine identification HPLC method	The retention time of the main peak of lamivudine corresponds to the standard	Positive	Positive	Positive
Lamivudine content HPLC method	The amount found must not be less than 90.0% or more than 110.0% of the declared amount.	154.1 mg/tab rec (102.7%)	150.6 mg/tab rec (100.4%)	148.9 mg/tab rec (99.3%)
Comparison of the percentages of the API content of the two products	The difference between the obtained percentages should not exceed 5%.	0%	2.3%	3.4%
Lamivudine dilution UV-Vis method	Not less than 80% (Q) of the declared amount of lamivudine	M1 = 102% M2 = 103% M3 = 102% M4 = 103% M5 = 101% M6 = 103%	M1 = 101% M2 = 105% M3 = 100% M4 = 99% M5 = 100% M6 = 97%	M1 = 101% M2 = 100% M3 = 99% M4 = 101% M5 = 99% M6 = 100%
Content uniformity by weight variation	AV < 15.0%	M1 = 104.9% M2 = 101.2% M3 = 102.2% M4 = 100.2% M5 = 102.6% M6 = 103.2% M7 = 101.2% M8 = 104.9% M9 = 104.1% M10= 102.6% AV= 5.0%	M1 = 98.5% M2 = 100.3% M3 = 99.1% M4 = 100.9% M5 = 100.8% M6 = 99.1% M7 = 101.5% M8 = 101.0% M9 = 104.2% M10= 100.3% AV= 2.9%	M1 = 101.8% M2 = 98.6% M3 = 104.1% M4 = 98.6% M5 = 101.7% M6 = 104.2% M7 = 101.8% M8 = 98.5% M9 = 104.2% M10 = 98.6% AV= 5.9%

a USP 42 specifications

HPLC: High Performance Liquid Chromatography, mg/tab rec: milligrams per coated tablet, API: active pharmaceutical ingredient, (Q): percentage of drug dissolved at time “t” (according to USP 42), AV: average variation (average variation between samples)

### Dissolution profiles

**Figure 1:**
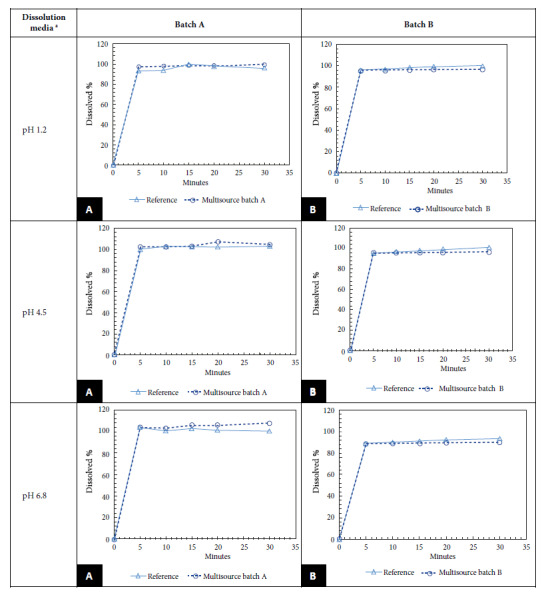
Dissolution profiles of lamivudine tablets (150 mg) of multisource A and B versus innovator in the three dissolution media.

We found that the dissolution profile results of LMV batches A and B were between 98.6% and 96.2% for pH 1.2; between 103.4% and 96.3% for pH 4.5 and between 106.1% and 89.5% for pH 6.8; at 15 min the percent dissolution in both cases was greater than 85%. Since both batches passed the *in vitro* test, it was not necessary to calculate the f2 (Figure 1). All samples, both multisource and innovative, met the WHO requirements for dissolution profiling. The difference between the percentage of active ingredient content of the reference product and the MDs was no more than 5%. Figure 1 shows the dissolution profiles of the LMV MDs marketed in Peru versus the data of the reference product.

## DISCUSSION

Evaluating the biopharmaceutical quality of generic antivirals is a strategy to provide adherence to antiretroviral treatment in HIV patients and to reduce resistance to this drug with effective therapy ^14^. In this study all samples, both the innovator LMV drug and both batches of LMV MD, met pharmacopeial quality requirements for identification, content, dose uniformity and dilution. In addition, the two multisource drug batches A and B showed similarity between the percentages of drug recovered with the innovator drug at all three physiological pHs and the percentage of dissolution was greater than 85% at 15 min in both cases, so it was not necessary to calculate the f2.

In contrast to our results regarding quality, Fernandes *et al*. reported that two generic batches of LMV failed the quality test for dissolution ^15^. Later in 2015, Wang *et al*. reported that 6 of 88 samples (from developing countries and internet purchases) failed the dissolution test at 1.5-8.3%, below the standard range in a multicenter study ^16^.

Dissolution testing is a powerful quality tool associated with drug absorption and drug bioavailability. Problems associated with poor quality drugs lead to subtherapeutic treatments, adverse effects, morbidity, mortality, and drug resistance ^4^.

Regarding the dissolution profiles, our results agree with the study conducted in Turkey by Otzurk *et al*. which obtained more than 85% of the labeled amount dissolved in 15 min in the three-dissolution media: pH 1.2 (97.5%, 100.0% and 99.9%); pH 4.5 (96.4%, 101.0% and 99.8%); and pH 6.8 (103.0%, 98.8% and 100.0%); therefore, the mathematical comparison of similarity (f2) was not necessary either ^17^. On the contrary, a study carried out in Argentina showed that the results obtained for LMV and abacavir presented an f2 greater than 50 for the three generic lots for both LMV and abacavir at pH 1.2 and 6.8; however, for pH 4.5, lot 1 did not meet f2 for either of the two actives ^18^.

The similarity factor is a measure of the percent dissolution (%) between the two curves. Dissolution profiles are considered similar if the value of f2 is ≥50. However, when 85% or more of the stated amount of the test or reference drugs dissolve in ≤15 min using the recommended dissolution media (pH 1.2, pH 4.6, and pH 6.8), they are considered similar dissolution profiles without requiring any mathematical calculation ^7^.

A study in Brazil found that batches from laboratory G (reference) showed similarity among themselves with fast dissolution G1, G2 and G3 (97.9%; 101.8% and 99.4%); while laboratory A and B drugs showed differences among their batches A1, A2 and A3 (103.6%; 100.3% and 100.6%) and B1, B2 and B3 (98.8%; 100.7% and 101.2%), however, at the end of the determined time, they reached a performance similar to that of innovator G, being considered pharmaceutical equivalents to the reference drug. On the contrary, the batches of laboratory C were not similar to each other C1, C2 and C3 showing an alarmingly low dissolution 73.9%; 7.2% and 11.7% ^11^.

A thorough evaluation of excipient similarity is critical and must be well established to demonstrate interchangeability (bioexcitation) ^4^. LMV exhibits characteristics of high solubility with the intrinsic dissolution method ^19^. One study presented the permeability of LMV as an active pharmaceutical ingredient (API) of high permeability (Class I) ^20^, but it is classified as class III of low permeability because it has an elimination rate as unchanged drug via the kidney of approximately 70%, characteristic of this class of API.

The strength of this study lies in the fact that LMV is the first drug of the HAART program of MINSA to be evaluated in Peru. As for the limitations of this study, it was difficult to obtain the sample, and it was not feasible to obtain the qualitative-quantitative composition of the innovative drug and the MDs to evaluate the excipients. Solubility and permeability studies were not performed because they were not within the objectives of the study as requested by the WHO and the DS 024-2018 SA.

The procurement of drugs for MINSA’s health strategy programs guarantees access to these products for the population; however, they must be of proven quality, safety and efficacy, otherwise we will find ourselves immersed in a loss of confidence in the health systems and a waste of financial resources. It is important to consider these tests: quality and dissolution kinetics, as requirements in the purchasing process, even more so if it is an antiviral drug.

In conclusion, we found that LMV innovator, LMV MD A and LMV MD B met the pharmacopeial quality specifications and demonstrated similar dissolution profiles to the innovator *in vitro*, which shows that LMV 150 mg tablets have *in vitro* equivalence. It is recommended to continue implementing drug interchangeability studies so that the population has greater access to safe, quality and affordable MDs.
