# Supplementary material for: Comparative evaluation of dissolution profiles of the generic drug lamivudine 150 mg tablet marketed in Peru vs. the innovative Epivir
Source: Rev Peru Med Exp Salud Publica. 2024 Mar 25;41(1):69–75. doi: 10.17843/rpmesp.2024.411.12821 (PMC11152243; doi:10.17843/rpmesp.2024.411.12821)
Supplement: Supplementary material. — Available in the electronic version of the RPMESP. [file rpmesp-41-01-12821-s001.pdf]

Material Suplementario

Anexo 1. Tabla de Estándares y reactivos usados en el estudio

Estándares	
Estándar de Lamivudina (secundario)	Sigma Aldrich
Estándar Lamivudina Resolución mezcla B USP (primario)	(United States Pharmacopeia)
**Reactivos**	Grado analítico. Todos los reactivos fueron de calidad ACS (American Chemical Society).
• Metanol grado HPLC<br>• ácido fosfórico<br>• y acetato de amonio	(J.T. Baker, México)
• Fosfato de potasio monobásico,<br>• ácido clorhídrico,<br>• cloruro de sodio, hidróxido de sodio	(Merck KGaA, Darmstadt, Germany)
• Acetato de sodio trihidratado	(Bio Basic)
• Ácido acético glacial	(Fisher)
• Agua grado HPLC (18,2 MΩ)	obtenida a través de un equipo purificador de agua Milli Q (Millipore Advantage A10).
