# Supplementary material for: Comparative evaluation of dissolution profiles of the generic drug lamivudine 150 mg tablet marketed in Peru vs. the innovative Epivir
Source: Rev Peru Med Exp Salud Publica. 2024 Mar 25;41(1):69–75. doi: 10.17843/rpmesp.2024.411.12821 (PMC11152243; doi:10.17843/rpmesp.2024.411.12821)
Supplement: Translation

# Translation: Evaluación comparativa de perfiles de disolución del medicamento genérico lamivudina tableta 150 mg comercializado en Perú frente al innovador Epivir

**Authors:** Malena Castañeda-Alarcón, Encarna García-Montoya, Javier Rodríguez-Calzado, María Flores-Rodríguez, Miguel Grande-Ortíz, Luis Moreno-Exebio

**Affiliations:** 1 Centro Nacional de Control de Calidad, Instituto Nacional de Salud, Lima, Perú.; 2 Departamento de Farmacia y Tecnología Farmacéutica y Físico Química, Facultad de Farmacia y Ciencias de la Alimentación, Universidad de Barcelona, Barcelona, España.

**Keywords:** Equivalencia Terapéutica, Medicamentos Genéricos, Lamivudina, Biofarmacéutica, Medicamentos Bioequivalentes, Disolución, VIH

## Abstract

La lamivudina es uno de los medicamentos más prescritos en el mundo, se utiliza para tratar la inmunodeficiencia humana y la hepatitis B. El objetivo del estudio fue evaluar los atributos de calidad y comparar los perfiles de disolución de dos lotes (A y B) del medicamento genérico lamivudina 150 mg tabletas con el medicamento innovador Epivir 150 mg tabletas. Se realizó un estudio analítico, experimental y de corte transversal, se usó un método espectrofotométrico a una longitud de onda de máxima absorción (λ) correspondiente a 270 nm, para medir el porcentaje de fármaco disuelto. El estudio evaluó identificación, contenido, disolución y uniformidad de masas. Se usó el aparato 2 USP (Paleta) 75 rpm, 900 mL de medio de disolución (37 ± 0,5 °C) a en tres medios de disolución: pH 1,2; 4,5 y 6,8. Se retiraron muestras de 5 mL a los 5, 10, 15, 20 y 30 min. Se encontró que ambos lotes de lamivudina genérico (A y B) presentan el mismo perfil cinético de disolución que el medicamento innovador. Ambas formulaciones cumplen con el criterio de medicamentos de disolución muy rápida (85% disuelto en 15 min), y de disolución rápida (85% disuelto en 30 min). Por lo tanto, no fue necesario calcular el factor de similitud. Se concluye que los medicamentos genéricos A y B son equivalentes *in vitro* con el medicamento innovador Epivir.

## INTRODUCCIÓN

La infección por el virus de la inmunodeficiencia humana (VIH) es un importante problema de salud pública en el mundo y el tratamiento antirretroviral evita las enfermedades relacionadas con el VIH. En América Latina, sólo el 65% de los casos reciben tratamiento; en el Perú ^1^^,^^2^^)^ el Programa Nacional de Terapia Antirretroviral de Gran Actividad (TARGA) a través del Ministerio de Salud (MINSA) del Perú trabaja en el control de la epidemia del VIH mediante estrategias de prevención, brindando gratuitamente el tratamiento, asegurando la disponibilidad de la medicación y la adherencia a la misma ^3^.

Asegurar que los medicamentos cumplan con los estándares de calidad es obligatorio para garantizar la seguridad y la eficacia del fármaco. Un medicamento genérico o multifuente (MM) es farmacéuticamente equivalente (alternativo) y puede, o no, ser terapéuticamente equivalente al innovador (referencia). Solo un MM que ha demostrado equivalencia terapéutica al innovador (*in vivo* o *in vitro*) es intercambiable ^4^. En la Unión Europea y los Estados Unidos de Norteamérica la equivalencia terapéutica es un requisito reglamentario para el registro de medicamentos genéricos ^5^.

En México, Brasil y Argentina se comercializan genéricos intercambiables, sin embargo, en Bolivia, Honduras, Nicaragua, República Dominicana y otros países como el Perú comercializan, aun, productos farmacéuticos sin estudios de equivalencia terapéutica ^6^. En el Perú para el registro, inscripción y reinscripción de medicamentos, la autoridad reguladora solicita estudios de intercambiabilidad mediante el Decreto Supremo 024-2018-SA, el cual se viene aplicando en el país de manera gradual o progresiva, en su primer listado estuvo el fármaco lamivudina (LMV) tabletas para la que se acepta estudios *in vitro* (bioexcencion) ^(^^7^.

Un medicamento debe cumplir los requisitos farmacopeicos para demostrar su calidad, entre ellos la prueba de disolución que determina el porcentaje (%) disuelto de fármaco en un solo punto. Para demostrar equivalencia terapéutica *in vitro*, además de la solubilidad y permeabilidad del fármaco, se evalúa la similitud de los perfiles de disolución (disuelto en varios puntos), debiendo presentar el MM un comportamiento semejante al innovador. Sin embargo, en el mundo existen informes ocasionales de medicamentos genéricos de calidad inferior ^(^^8^. En el Perú se encontraron medicamentos genéricos que no cumplieron la calidad requerida, entre ellos, algunos lotes de metformina clorhidrato 850 mg ^9^, moxifloxacino tabletas 400 mg ^10^no pasaron la prueba de similitud de perfiles de disolución. Por esta razón es imperativo investigar la calidad de los medicamentos dispensados en el país, en este caso LMV, fármaco para el tratamiento de VIH dispensado en el programa TARGA del MINSA.

El objetivo del estudio fue evaluar la calidad y comparar la similitud de la cinética de disolución de dos lotes del MM LMV tabletas 150 mg, del programa TARGA del MINSA, a través de los perfiles de disolución comparándolas con el fármaco innovador (referencia) Epivir.

**MENSAJE CLAVE**

Motivación para realizar el estudio: Evaluar la calidad de los medicamentos antirretrovirales usados en el tratamiento del VIH dispensados en el Programa TARGA del Ministerio de Salud de Perú.

Principales hallazgos: Se encontró que dos lotes de medicamentos genéricos de lamivudina alcanzaron un porcentaje de disolución mayor del 85% a los 15 min, siendo equivalentes *in vitro* al producto de referencia Epivir.

Implicancias: Existe la necesidad de aplicar la normatividad vigente respecto a equivalencia entre fármacos por parte de la autoridad regulatoria previo a su autorización e incluir ensayos de perfil de disolución como requisito en las compras públicas de medicamentos, especialmente en las estrategias nacionales (VIH, TBC, etc.), con la finalidad de asegurar productos de calidad para la población.

## EL ESTUDIO

La relación de sustancias químicas y reactivos están disponibles en el material suplementario.

### Tipo de estudio y muestras

El estudio fue experimental, se realizó en el Servicio de Desarrollo de Medicamentos (SDM) de la Universidad de Barcelona, y en el Centro Nacional de Control de Calidad (CNCC) del INS, la muestra estuvo constituida por 200 tabletas del medicamento innovador de LMV de 150 mg y 200 tabletas de 150 mg LMV MM del lote A y B dispensados por el programa TARGA del MINSA en el 2019 (Tabla 1). 

**Tabla 1:** Información de manufactura de los fármacos evaluados.

Información	Principio activo		
	Referencia	Lote A	Lote B
	Epivir (lamivudina)	Lamivudina	Lamivudina
Cantidad declarada	150 mg	150 mg	150 mg
Presentación	Comprimidos	Comprimido oral	Comprimido oral
Lote	275 P	E180608	E182268
País de origen	Reino unido	India	India
Fabricante	Glaxo	Hetero Labs Limited	Hetero Labs Limited
Fecha de vencimiento	10-2021	03-2020	10-2020

### Validación del método y calibración de equipos

Se validó el perfil de disolución mediante método espectrofotométrico. Se consideró los siguientes parametros: linealidad, precisión, exactitud, estabilidad e influencia del filtro, según United States Pharmacopeia (USP 42) Conferencia Internacional de Armonización (ICH) ^12^^,^^13^. Para la linealidad se preparó una curva de calibración para siete concentraciones en el rango de 10% a 120% del estándar (St) de LMV. Para la exactitud se analizaron soluciones St LMV al 10%, 60%, y 120%. Para la precisión se analizaron seis réplicas (n=6) al 100% del LMV St y se calculó la desviación estándar relativa (DSR). Para la influencia del filtro se usó 100% de LMV St se utilizó filtros de diferentes materiales y tamaños de poro. Para la estabilidad se leyó solución LMV St al 100% y se almacenó 2 a 8 ºC durante 24 h, luego se midió nuevamente su concentración. Todas las muestras se leyeron por triplicado a λ = 270 nm en los tres medios de disolución (pH: 1,2; 4,5 y 6,8).

### Ensayos de calidad

Los ensayos de identificación, contenido, disolución y uniformidad de LMV fueron realizados según la USP 42, capítulos generales <197M>, <621>, <711> y <905>. La identificación del fármaco se realizó por el método de espectrometría Infrarrojo (IR) y por cromatografía líquida de alta resolución (CLAR) ultravioleta visible (UV-VIS). El ensayo de contenido por CLAR - UV-VIS con detector UV 277 nm; columna L1 25cm × 4,6 mm (5 um), volumen 20 uL, velocidad de flujo 1mL/min y temperatura de columna de 30 +/- 5 °C. Disolución por espectrometría UV-VIS y la uniformidad de unidades de dosificación se realizó por variación de pesos ^12^. 

### Perfil de disolución

Se midió la cantidad de fármaco liberado (porcentaje de la cantidad etiquetada). Se analizaron 6 y 12 comprimidos para los lotes A, B y el innovador, las muestras fueron tomadas manualmente con reposición (10 mL cada vez) a 5, 10, 15, 20 y 30 min; posteriormente 1 mL de las alícuotas tomadas de cada muestra se aforaron a 10 mL en los tres medios de disolución, se filtraron con PVDF 0,45 µm y se leyeron las absorbancias a λ = 270 nm por el método espectrofotométrico UV-VIS ^4^^,^^12^. Todos los medios se prepararon sin enzimas y se desgasificaron al vacío con agitación mecánica. Las condiciones del ensayo fueron aparato 2 (paleta) a 75 rpm y 900 mL de volumen cada medio de disolución a 37 ± 0,5 °C ^12^. Los equipos utilizados para los perfiles de disolución fueron disolutor Erweka® DT 700 para 8 vasos y espectrofotómetro Specord 205 Analitikjena, disolutor Hanson Vision Elite G2 Heather para 8 vasos y espectrofotómetro Janson V-650 Spectrophotometer.

### Análisis estadístico

Se utilizó Microsoft Excel 2019 como herramienta principal. Para potenciar la funcionalidad y precisión de las evaluaciones, se incorporaron complementos especializados, entre ellos, «herramientas para análisis» que proporcionaron funcionalidades avanzadas en estadística. Se uso DDSolver, un complemento de Excel, para el cálculo del factor de similitud entre los perfiles de disolución de medicamentos (f2), no obstante, no se aplicó debido a que los medicamentos se disolvieron en más del 85% en menos de 15 min. La cantidad disuelta de los principios activos se llevó a cabo mediante gráficas de dispersión, considerando cada lote y el pH correspondiente a los perfiles de disolución. Para los parámetros de validación se aplicó estadística descriptiva como el cálculo de la media aritmética, desviación estándar y desviación estándar relativa. 

## HALLAZGOS

Los resultados de la validación demuestran que el método es lineal en el rango de concentración de 0,0170 a 0,2037 mg/mL para todos los medios de disolución. Los valores obtenidos para la desviación estándar relativa (DSR) y la recuperación de las muestras de solución estándar indican que la precisión y la exactitud del método es satisfactoria. Se seleccionó el tipo de filtro PVDF 0,45 μm porque presentó mayor % de recuperación entre los filtros evaluados, y se determinó que el método era estable a las 24 h bajo condiciones de refrigeración (2 a 8 °C) (Tabla 2).

**Tabla 2:** Validación del Método de perfiles de disolución.

Parámetros	Especificaciones ^a^	Resultados		
		pH=1,2 ^b^	pH=4,5 ^c^	pH=6,8 ^d^
Linealidad	r no menor de 0,999	1,0000	0,9997	1,0000
	CV % menor o igual a 2%	0,366%	1,521%	0,478%
	Intercepto	0,0022	- 0,0017	0,0006
Precisión	DSR % menor o igual a 2%	0,03%	0,06%	0,02%
Exactitud	% de recuperación 95-105%	102,33%	103,13%	100,41%
	DSR % menor o igual a 2%	0,3882%	0,9575%	0,4077%
Estabilidad	% de recuperación 98-102%	98,36%	98,80%	99,69%
Influencia del filtro ^e^	% de recuperación 98-102%	99,05%	98,63%	94,27%

a Criterio de aceptación, según ICH Validation Of Analytical Procedures: Text And Methodology Q2(R1)

b Fluido gástrico simulado pH 1,2

c Buffer acetato pH 4,5

d Fluido intestinal simulado pH 6,8

e El filtro PVDF 0,45 μm presentó mayor % de recuperación

r: coeficiente de correlación de Pearson

CV %: coeficiente de variación del factor de respuesta, DSR %: desviación estándar relativa

### Control de calidad de medicamentos

Se encontró que el medicamento innovador y los dos lotes (A y B) de MM pasaron las pruebas de calidad: identificación, contenido, disolución y uniformidad de masas, según lo requerido en la USP 42. Para la identificación del innovador y los MM Lote A y B, los cromatogramas mostraron que los tiempos de retención se corresponden con la solución estándar de LMV. El contenido de todas las muestras se encontró dentro de 90 a 110%. Para las pruebas de disolución el porcentaje de recuperación del innovador fluctuó entre 102 y 103%, para los MM lote A entre 97 y 105% y para el MM B entre 99 y 101%. Todos cumplieron la especificación de disolución ≥ 80% de la cantidad etiquetada. La uniformidad de todas las muestras estuvieron dentro del rango de aceptación de la USP 42 (Tabla 3).

**Tabla 3:** Resultados de control de calidad del innovador (referencia), lote A y B de lamivudina.

Ensayos	Especificaciones ^a^	Resultados		
		Referencia	Multifuente Lote A	Multifuente Lote B
Identificación de lamivudina Método HPLC	El tiempo de retención del pico principal de lamivudina se corresponde al estándar	Positivo	Positivo	Positivo
Contenido de lamivudina Método HPLC	La cantidad hallada no debe ser menor a 90,0% ni mayor a 110,0% de la cantidad declarada	154,1 mg/tab rec (102,7%)	150,6 mg/tab rec (100,4%)	148,9 mg/tab rec (99,3%)
Comparación de los porcentajes del contenido del IFA de ambos productos	La diferencia entre los porcentajes obtenidos no debe ser mayor a 5%	0%	2,3%	3,4%
Disolución de lamivudina Método UV-Vis	No menos de 80% (Q) de la cantidad declarada de lamivudina	M1 = 102% M2 = 103% M3 = 102% M4 = 103% M5 = 101% M6 = 103%	M1 = 101% M2 = 105% M3 = 100% M4 = 99% M5 = 100% M6 = 97%	M1 = 101% M2 = 100% M3 = 99% M4 = 101% M5 = 99% M6 = 100%
Uniformidad de contenido por variación de pesos	AV < 15,0%	M1 = 104,9% M2 = 101,2% M3 = 102,2% M4 = 100,2% M5 = 102,6% M6 = 103,2% M7 = 101,2% M8 = 104,9% M9 = 104,1% M10= 102,6% AV= 5,0%	M1 = 98,5% M2 = 100,3% M3 = 99,1% M4 = 100,9% M5 = 100,8% M6 = 99,1% M7 = 101,5% M8 = 101,0% M9 = 104,2% M10= 100,3% AV= 2,9%	M1 = 101,8% M2 = 98,6% M3 = 104,1% M4 = 98,6% M5 = 101,7% M6 = 104,2% M7 = 101,8% M8 = 98,5% M9 = 104,2% M10 = 98,6% AV= 5,9%

a Especificaciones de la USP 42

HPLC: High Performance Liquid Chromatography, mg/tab rec: miligramos por tableta recubierta, IFA: ingrediente farmacéutico activo, (Q): porcentaje de fármaco disuelto a un tiempo “t” (según USP 42), AV: average variation (variación promedio entre las muestras)

### Los perfiles de disolución

Los resultados de perfil de disolución de los lotes A y B de LMV de nuestro estudio estuvieron entre el 98,6% y 96,2% para el pH 1,2; entre 103,4% y 96,3% para el pH 4,5 y entre 106,1% y 89,5% para el pH 6,8. Se observó que a los 15 min el porcentaje de disolución en ambos casos fue mayor del 85%. Como los dos lotes pasaron la prueba *in vitro*, no fue necesario calcular el f2. (Figura 1). Todas las muestras tanto multifuentes como innovador cumplieron los requisitos de la OMS para ejecución de perfiles de disolución. La diferencia entre el porcentaje del contenido del principio activo del producto de referencia y los MM no fue mayor al 5%. La Figura 1 muestra los perfiles de disolución de los LMV MM comercializados en Perú versus los datos del producto de referencia. 

**Figura 1:** Perfiles de disolución de lamivudina tabletas (150 mg) de los multifuentes A y B frente al innovador en los tres medios de disolución.

## DISCUSIÓN

Evaluar la calidad biofarmacéutica de los antivirales genéricos es una estrategia para aportar adherencia al tratamiento antirretroviral en pacientes con VIH y lograr reducir la resistencia a este fármaco con una terapia eficaz ^14^. En este estudio todas las muestras tanto el medicamento LMV innovador y ambos lotes de LMV MM cumplieron requisitos de calidad farmacopeicas para identificación, contenido, uniformidad de dosis y disolución. Además, los dos lotes de medicamentos multifuentes A y B demostraron similitud entre los porcentajes de fármaco recuperado con el medicamento innovador, en los tres pH fisiológicos y se observó que a los 15 min el porcentaje de disolución en ambos casos fue mayor del 85%, por lo que no fue necesario calcular el f2. 

En contraste con los resultados de calidad encontrados en esta investigación, Fernandes *et al*. informaron que dos lotes genéricos de LMV no cumplieron la prueba de calidad para disolución ^15^. Posteriormente en 2015, Wang *et al*. reportaron que 6 de 88 muestras (de países en vías de desarrollo y compras por Internet) fallaron la prueba de disolución en 1,5-8,3%, por debajo del rango estándar en un estudio multicéntrico ^16^.

La prueba de disolución es una poderosa herramienta de calidad asociada a la absorción del fármaco y la biodisponibilidad del mismo. Los problemas relacionados con los medicamentos de baja calidad conllevan a tratamientos subterapeuticos, efectos adversos, morbilidad, mortalidad, y resistencia a los medicamentos ^4^. 

En relación a los perfiles de disolución nuestros resultados coinciden con el estudio realizado en Turquía por Otzurk *et al*. que obtuvo más del 85% de la cantidad etiquetada disuelto en 15 min en los tres medios de disolución: pH 1,2 (97,5%, 100,0% y 99,9%); pH 4,5 (96,4%; 101,0% y 99,8%); y pH 6,8 (103,0%; 98,8% y 100,0%); por lo tanto, la comparación matemática de similitud (f2) tampoco fue necesaria ^17^. Por el contrario, un estudio realizado en Argentina demostró que el resultado obtenido para LMV y abacavir presentaron un f2 mayor a 50 para los tres lotes genéricos tanto para LMV como para abacavir en los pH 1,2 y 6,8; sin embargo, para el pH 4,5 el lote 1 no cumplió f2, para ninguno de los dos activos ^18^. 

El factor de similitud es una medida del porcentaje de disolución (%) entre las dos curvas. Los perfiles de disolución se consideran similares si el valor de f2 es ≥50. Sin embargo, cuando el 85% o más de la cantidad declarada de los medicamentos de ensayo o de referencia se disuelven en ≤15 min usando los medios de disolución recomendados (pH 1,2, pH 4,6 y pH 6,8), se consideran perfiles de disolución similares sin requerir ningún cálculo matemático ^7^.

Un estudio en Brasil encontró que lotes del laboratorio G (referencia) mostraron similitud entre sí con disolución rápida G1, G2 y G3 (97,9%; 101,8% y 99,4%); mientras que los medicamentos laboratorio A y B mostraron diferencias entre sus lotes A1, A2 y A3 (103,6%; 100,3% y 100,6%) y B1, B2 y B3 (98,8%; 100,7% y 101,2%), sin embargo, al final del tiempo determinado alcanzaron un rendimiento similar al del innovador G, siendo considerados equivalentes farmacéuticos al fármaco referencia. Por el contrario, los lotes del laboratorio C no resultaron similares entre sí C1, C2 y C3 mostrando una alarmante baja disolución 73,9%; 7,2% y 11,7% ^11^. 

Una evaluación exhaustiva de la similaridad de los excipientes es crítica y debe ser bien establecida, para demostrar intercambiabilidad (bioexcencion) ^4^. LMV presenta características de una alta solubilidad, con el método de disolución intrínseca ^19^. Un estudio presentó la permeabilidad de LMV como un ingrediente farmacéutico activo (IFA) de alta permeabilidad (Clase I) ^20^, pero se clasifica como clase III de baja permeabilidad porque presenta una eliminación como fármaco inalterado vía renal de aproximadamente 70%, característica de esta clase de IFA.

La fortaleza de este estudio radica que LMV es el primer fármaco del programa TARGA del MINSA evaluado en Perú. En cuanto a las limitaciones, se presentó dificultad para conseguir la muestra, además no fue factible obtener la composición cuali-cuantitativa del medicamento innovador y los MM para realizar la evaluación de excipientes, no se realizaron los estudios de solubilidad y permeabilidad porque no estuvieron dentro de los objetivos del estudio tal como lo solicita la OMS y el DS 024-2018 SA.

La adquisición de medicamentos para los programas de las estrategias sanitarias del MINSA garantiza el acceso a la población de estos productos; sin embargo, estos deben presentar calidad, seguridad y eficacia comprobada, de lo contrario nos encontraremos inmersos en una pérdida de confianza en los sistemas de salud y desperdicio de recursos financieros. Es importante que se consideren estas pruebas: calidad y cinética de disolución, como requisitos en los procesos de compra, más aún si se trata de un fármaco antiviral.

En conclusión, se encontró que LMV innovador, LMV MM A y LMV MM B cumplieron las especificaciones de calidad farmacopeicas y demostraron perfiles de disolución similares al innovador *in vitro*, lo cual muestra que LMV tabletas de 150 mg tienen equivalencia *in vitro*. Se recomienda continuar implementando estudios de intercambiabilidad de medicamentos a fin de que la población tenga un mayor acceso a MM seguros, de calidad y a costo accesible.
